# Thermally activated delayed fluorescence with 7% external quantum efficiency from a light-emitting electrochemical cell

**DOI:** 10.1038/s41467-019-13289-w

**Published:** 2019-11-22

**Authors:** Petter Lundberg, Youichi Tsuchiya, E. Mattias Lindh, Shi Tang, Chihaya Adachi, Ludvig Edman

**Affiliations:** 1 The Organic Photonics and Electronics Group, Umeå University, SE-901 87 Umeå, Sweden; 2 Center for Organic Photonics and Electronics Research (OPERA), Kyushu University, 744 Motooka, Nishi-ku, Fukuoka 819-0395 Japan; 3 JST, ERATO, Adachi Molecular Exciton Engineering Project, Kyushu University, 744 Motooka, Nishi-ku, Fukuoka 819-0395 Japan; 4 LunaLEC AB, Linnaeus väg 24, SE-901 87 Umeå, Sweden; 5 Department of Chemistry and Biochemistry, Kyushu University, 744 Motooka, Nishi-ku, Fukuoka 819-0395 Japan; 6 International Institute for Carbon Neutral Energy Research (WPI-I2CNER), Kyushu University, 744 Motooka, Nishi-ku, Fukuoka 819-0395 Japan

**Keywords:** Electronic devices, Organic LEDs, Electronic and spintronic devices

## Abstract

We report on light-emitting electrochemical cells, comprising a solution-processed single-layer active material and air-stabile electrodes, that exhibit efficient and bright thermally activated delayed fluorescence. Our optimized devices delivers a luminance of 120 cd m^−2^ at an external quantum efficiency of 7.0%. As such, it outperforms the combined luminance/efficiency state-of-the art for thermally activated delayed fluorescence light-emitting electrochemical cells by one order of magnitude. For this end, we employed a polymeric blend host for balanced electrochemical doping and electronic transport as well as uniform film formation, an optimized concentration (<1 mass%) of guest for complete host-to-guest energy transfer at minimized aggregation and efficient emission, and an appropriate concentration of an electrochemically stabile electrolyte for desired doping effects. The generic nature of our approach is manifested in the attainment of bright and efficient thermally activated delayed fluorescence emission from three different light-emitting electrochemical cells with invariant host:guest:electrolyte number ratio.

## Abstract (web summary)

Thermally activated delayed fluorescence (TADF) emitters deliver impressive performance in sophisticated multilayer devices. Here, the authors report efficient TADF emission from light-emitting electrochemical cells that feature a solution-processed active layer in between two air-stable electrodes.

## Introduction

Emerging emissive technologies—which are thin, flexible and emit from a large-area—are projected to open up for new functionalized applications in, e.g., medicine^1,2^, security^3^, communication^4^, and packaging^5^. The light-emitting electrochemical cell (LEC) is such an area-emissive device that can be thin and flexible^6–10^, and recent studies have demonstrated that well-designed LECs, in addition, can be highly efficient at significant luminance^11^, and be fabricated with low-cost and scalable solution-based methods^12–15^. However, for several of the projected high-volume applications it is further desirable if the constituent device materials are sustainable^16–18^, and it is therefore unfortunate that current efficient LECs and organic light-emitting diodes (OLEDs) commonly employ emitters that comprise very rare and expensive metals from the platinum group in the periodic table^19–21^.

It is in this context that the recent introduction of high-efficiency metal-free organic emitters, which feature thermally activated delayed fluorescence (TADF), is particularly relevant^22^. In brief, by a rational molecular design, the lowest excited singlet state (S_1_) of a TADF-active emitter is positioned close in energy to its lowest excited triplet state (T_1_), so that a reverse intersystem crossing (RISC) from T_1_ to S_1_ can be thermally promoted at room temperature. The desired consequence is that both singlet and triplet excitons (i.e., all of the electrically generated excitons) can be harvested for light emission via prompt and delayed fluorescence, respectively, from the S_1_ state.

TADF-active emitters are increasingly being implemented in OLEDs^23^, and current state-of-the-art TADF-OLEDs feature luminance values well above 1000 cd m^−2^ for a wide range of emission colors, as well as high efficiencies, as quantified by reported external quantum efficiencies (EQEs) exceeding 30%^23–27^. It should, however, be noted that the roll-off for TADF-OLEDs commonly is significant^28^, and that TADF-OLEDs typically comprise a vacuum-deposited multilayer active material and a cathode that is unstable under ambient air, with the latter translating into a relatively expensive fabrication process.

The LEC is formally distinguished from the OLED by the presence of mobile ions in the active material^29–36^. It is the redistribution of these ions during operation that renders the LEC robust to variations in thickness of the active material and allows for a simple and air-stabile device structure, comprising a single-layer active material and air-stabile electrodes^37–39^, which in turn makes the LEC uniquely fit for low-cost and high-volume solution-based processing^40^. At the same time, the LEC exhibits additional material requirements over the OLED: notably a balanced electrochemical doping capacity^41^, compatibility of the electrolyte with the other active-material constituents for small phase separation^42^, and sufficient electrochemical stability to suppress side reactions^43^; and the unfortunate fact is that the current state-of-the-art performance for TADF-LECs is modest^44–47^, with the highest EQE of 0.4% attained at a low luminance of 13 cd m^−2^ ^45,47^, while the peak luminance at constant-bias operation is 228 cd m^−2^ at an EQE of 0.1%^44^.

In this study, we present an active material that fulfills the specific requirements for LEC operation, and at the same time features efficient TADF emission. Specifically, the developed active material features a majority host blend, comprising a high-molecular-weight poly(9-vinylcarbazole) (PVK) polymer for p-type doping and robust solution processing and a 1,3-bis[2-(4-tert-butylphenyl)-1,3,4-oxadiazo-5-yl]benzene (OXD-7) small molecule for n-type doping, a TADF-active guest emitter at a concentration of 0.5–0.8 mass% for efficient host-to-guest energy transfer at minimized guest–guest aggregation, and an electrochemically stabile tetrahexylammonium tetrafluoroborate (THABF_4_) ionic liquid at a 3.8 mass% concentration for optimized electrochemical doping. We demonstrate that a yellow-emitting TADF-LEC, with the optimized single-layer active material sandwiched between two air-stabile electrodes and with 2-[4-(diphenylamino)phenyl]-10,10-dioxide-9H-thioxanthen-9-one (TXO-TPA) as the guest emitter, delivers a record-high EQE of 7.0% at a significant luminance of 120 cd m^−2^. By simply increasing the current density, it is possible to achieve a second-fast turn-on and peak luminance values well above 1000 cd m^−2^. We also demonstrate the generic nature of the design concept through the realization of two additional high-efficiency and high-luminance TADF-LECs, which emit cyan and orange light, and which comprise essentially the same ratio between host, guest, and electrolyte. We finally discuss the TADF-LEC operation in the context of different quenching reactions, and present a future design path towards a further improved device performance.

## Results

### Material selection and characterization

For TADF- and triplet-emitting active materials, it is a common practice to disperse the emitter as a minority guest into a larger energy-gap majority host matrix. This is done to efficiently limit the diffusion of the long-lived triplet excitons so that non-desired triplet-triplet quenching reactions can be suppressed or preferably eliminated^48^. This type of majority-host/minority-emissive-guest architecture is also attractive since it lowers self-absorption of the guest-generated light within the active material^47,49,50^, but a drawback is that the guest emitter can function as a trap for electron and hole transport, particularly so for thicker host-guest layers.

**Fig. 1:**
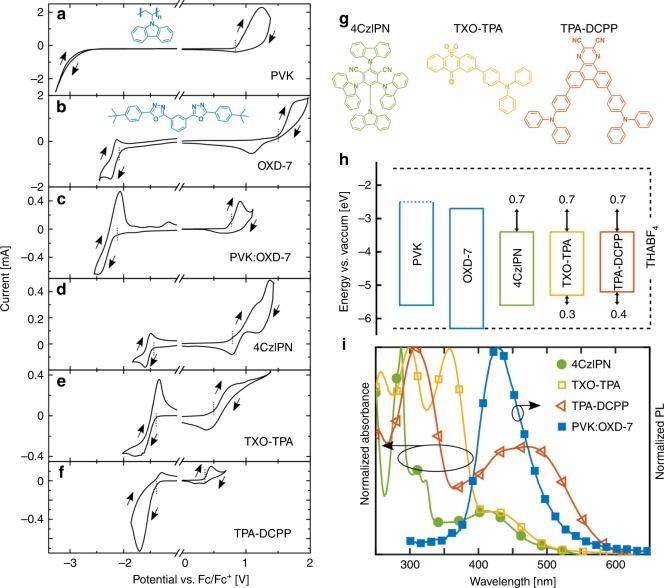
Electrochemical and optical characterization of the device materials. Voltammograms recorded on thin films of the host compounds (**a**, **b**), the blend host (**c**), and the guest compounds (**d**–**f**), as identified in the insets. The vertical dotted lines indicate the onset potentials for oxidation and reduction. The chemical structures of the host compounds are included as insets in (**a**) and (**b**), while the chemical structures of the guest compounds are displayed in (**g**). **h** The electron-energy levels of the host and guest compounds, as derived from the voltammograms and the absorption data. The electrochemical stability window of the THABF_4_ electrolyte is indicated by the dashed line. **i** The normalized absorbance of the guest compounds (left *y*-axis) and the normalized photoluminescence (PL) of the blend host (solid blue squares, right *y*-axis). The film thickness was 100 nm, and the guests were dispersed in polystyrene at 10 mass%. The PL excitation wavelength was 300 nm.

Here, we have selected a blend of the polymer PVK and the small molecule OXD-7 in a 1:1 mass ratio for the host matrix; the chemical structures of the two constituents in the blend host are displayed in the insets of Fig. 1a, b. We have investigated three potentially TADF-active compounds for the guest: the cyan/green-emitting 2,4,5,6-tetra(9H-carbazol-9-yl)isophthalonitrile (4CzIPN), the yellow-emitting TXO-TPA, and the orange-emitting 7,10-Bis(4-(diphenylamino)phenyl)-2,3-dicyanopyrazinophenanthrene (TPA-DCPP), and their chemical structures are displayed in Fig. 1g. For the electrolyte, we selected the THABF_4_ ionic liquid. We emphasize that the inclusion of a high-molecular-weight polymer (here, the PVK host) was found to be critical in order to allow for a robust and repeatable solution-based processing of the active material. We also note that the PVK:OXD-7 blend host offers balanced electron and hole transport which is highly beneficial for efficient LEC operation^11^.

The TADF emitters are designed to feature a weak spatial overlap between the highest occupied molecular orbital (HOMO) and the lowest unoccupied molecular orbital (LUMO), since this renders the energy difference between the S_1_ and T_1_ states small and makes sure that one prerequisite for RISC from the T_1_ state to the emissive S_1_ state is accomplished. Density functional theory calculations show that this design criterion is fulfilled for all three investigated compounds. Specifically, the LUMO of 4CzIPN is positioned on the central dicyanobenzene moiety, while its HOMO is delocalized over the four peripheral carboxyl groups^22^. For TXO-TPA the LUMO of is positioned on the 9-H-thioxanthen-9-one-10,10-dioxide (TXO) and the HOMO on the triphenylamine (TPA) moiety^28^. And, finally, TPA-DCPP features a LUMO on the 2,3-dicyanopyrazinophenanthrene (DCPP) moiety while the HOMO is localized on the two TPA units^51^.

A functional active material in an LEC is p-type-doped (oxidized) at the anode and n-type-doped (reduced) at the cathode, so that the light-emitting p–n junction can form in the bulk of the active material. In order to investigate whether this basic requirement is fulfilled, we utilized cyclic voltammetry (CV) to study the reduction and oxidation behavior of the constituents in the active material. Figure 1a–f presents CV traces for the two host constituents, the blend host, and the three guest emitters in thin-film form. We observe that OXD-7 (Fig. 1b) and the three guest emitters (Fig. 1d–f) exhibit significant and relatively reversible reduction and oxidation events, while PVK (Fig. 1a) only displays a significant reversible oxidation but no reversible reduction. This suggests that OXD-7 and the three guest emitters feature p-type and n-type doping capacity, while PVK only can be p-type doped. For the PVK:OXD-7 blend host (Fig. 1c), the CV data imply that PVK is (preferentially) p-type doped and OXD-7 is n-type doped, as concluded by a comparison with the CV traces for the pure PVK and OXD-7 films.

With the CV data at hand, we can calculate the HOMO and LUMO energy levels with the equation *E*_HOMO/LUMO_ (eV) = −(4.8 eV + ${\mathrm{e}}V_{{\mathrm{Fc}}/{\mathrm{Fc}}^ + }^{{\mathrm{ox}}/{\mathrm{red}}}$), using the measured onset potentials for oxidation and reduction^52^, as indicated by the dotted lines in Fig. 1a–f. The exception is the LUMO of PVK (because PVK cannot be electrochemical reduced), which was instead estimated from the absorption onset in Supplementary Fig. 1a and the HOMO level. During transport in a host:guest material, the guest can act as an electron/hole trap if it features a lower LUMO/higher HOMO than the host compound(s), and these trap energies can have a profound influence on both the transport and emission capacity of LEC (and OLED) devices^11^. Figure 1h and Supplementary Table 1 present the derived energy levels of all host and guest compounds, and the trap energies for electron and hole transport in the corresponding host:guest active materials. We find that both electrons and holes can be trapped on TXO-TPA and TPA-DCPP, while only electrons are trapped on 4CzIPN. It is further established that the electron trap is consistently deeper at −0.7 eV than the hole trap, which features a depth of −0.3 eV and −0.4 eV for TXO-TPA and TPA-DCPP, respectively.

The oxidation and reduction potentials of the THABF_4_ electrolyte were derived with CV to be positioned at −6.3 eV and −1.5 eV, respectively^11^, as indicated by the dashed lines in Fig. 1h. Since both the reduction and oxidation of the electrolyte takes place at a higher absolute potential than the blend host and the three guests, it is reasonable to anticipate that the THABF_4_ electrolyte, as desired, will remain electrochemically stabile during the electrochemical doping of the host and guest compounds^43^. Note that this stability requirement implies that the electrolyte ions do not participate in direct electrochemical redox transfer during LEC operation, but that they play a critical role in the electrostatic compensation of the redox charge introduced onto the host and guest compounds during the electrochemical doping reactions.

Figure 1i presents the absorption spectra of the three TADF guests dispersed in a poly(styrene) matrix at 10 mass% (left y-axis) and the photoluminescence (PL) of the PVK:OXD-7 blend host (right *y*-axis). The PL spectrum of the blend host (solid blue squares) peaks at 430 nm, which corresponds to the energy difference between the LUMO of OXD-7 and the HOMO of PVK of 2.9 eV (see Supplementary Table 1). This implies that the blend host features exciplex emission, with the electron located on OXD-7 and the hole on PVK. This conclusion is further supported by that the PL spectrum of the PVK:OXD-7 blend host is red-shifted with respect to the PL spectra of the pure PVK and OXD-7 constituents (see Supplementary Fig. 1b). This in turn implies that the two host constituents PVK and OXD-7 are well-blended on the nm-scale in the blend-host film, so that the excitons created following photon absorption have time to reach a PVK/OXD-7 interface before decaying radiatively. Importantly, the PL spectrum of the blend host in Fig. 1i significantly overlaps the absorbance spectrum of all three of the TADF guests, particularly that of the smallest-gap TPA-DCPP (open red triangles). This demonstrates that a principle requirement for Förster resonance energy transfer (FRET) from the host to guest is fulfilled for all three host:guest systems^53,54^.

The energy difference between T_1_ and S_1_ for an exciplex emitter is commonly only a few meV^55,56^, and since we above identified that the PVK:OXD-7 blend host features exciplex emission, we draw the conclusion that its triplet energy can be estimated by the energy difference between the LUMO of OXD-7 and the HOMO of PVK; i.e., the triplet level of the blend host is 2.9 eV. In this context, we note that the reported triplet energy for PVK is 3.0 eV^23^ and 2.7 eV for OXD-7^24^. We also remember that the energy difference between the singlet and triplet states for a TADF emitter by design is very small, which allows us to estimate the triplet energy of our three TADF emitters with the difference between the measured values for the LUMO and HOMO presented in Supplementary Table 1. We thus find that the triplet energy is 2.2 eV for 4CzIPN, 1.9 eV for TXO-TPA, and 1.8 eV for TPA-DCPP. As these values are invariably lower than the triplet energy of the PVK:OXD-7 blend host, the conclusion is that a host-to-guest energy transfer by the Dexter mechanism is plausible for all three host:guest systems^57^.

### Optimization of the active material and the LEC performance

**Fig. 2:**
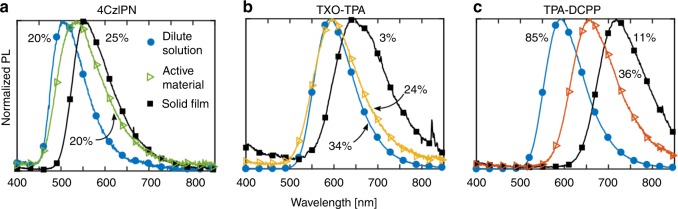
Influence of matrix on photoluminescence (PL) of guest emitter. The normalized PL spectra of the thermally activated delayed fluorescence guests 4CzIPN (**a**), TXO-TPA (**b**), and TPA-DCPP (**c**) in dilute 0.01 g L^−1^ toluene solution (solid blue circles), in the host-guest active material at 10 mass% guest concentration and with 4 mass% THABF_4_ (open triangles), and in a solid film (solid black squares). The value for the PL quantum yield (PLQY) is included next to the corresponding PL spectrum. The excitation wavelength was 300 nm.

Figure 2 presents the PL spectra, and the corresponding values for the PL quantum yield (PLQY, see inset next to the corresponding graph), of the three TADF guests in dilute toluene solution (0.01 g L^−1^, solid blue circles), as dispersed in a guest-rich LEC active material (10 mass% guest, 86 mass% host, 4 mass% electrolyte, open triangles), and in a solid pure film (solid black squares). All PL spectra are relatively broad with a full width at half maximum of ~60 nm. It is notable that no contribution from the blend host (PL spectrum spanning 400–460 nm, see Supplementary Fig. 1b) is visible in the PL from the three active materials (open triangles), implying a complete host-to-guest energy transfer. As expected, the largest energy-gap 4CzIPN featured the highest-energy (shortest wavelength) PL, the intermediate-gap TXO-TPA displayed the intermediate-energy PL, and the smallest-gap TPA-DCPP exhibited the lowest-energy PL.

The general trend is a red-shift of the PL spectrum from the guest when going from dilute solution, over the dispersed active material film, to the most concentrated pure solid film. The red-shift is most significant at ~120 nm for TPA-DCPP in Fig. 2c, and it is coupled with a very strong drop of the PLQY from 85% for the dilute solution, over 36% for the active material, to 11% in the solid film. A similar coupled (but weaker) red-shift in PL and drop in PLQY with increasing concentration was also observed for TXO-TPA in Fig. 2b, while 4CzIPN (Fig. 2a) featured the weakest red-shift at an essentially retained PLQY.

We attribute the red-shift in PL and the drop in PLQY to guest aggregation and guest conformational changes, which lower the efficiency of the radiative S_1_ → S_0_ transition and/or increase the T_1_ → S_1_ energy gap so that the RISC rate is slowed down. The smallest-gap TPA-DCPP features the highest “intrinsic” PLQY of 85% in “free and isolated” form (in dilute solution), but it also suffers from the most significant aggregation/conformation effects in the active material. In contrast, the intermediate-gap TXO-TPA and the largest-gap 4CzIPN exhibit lower intrinsic PLQY in isolated form, but are more resistant to detrimental aggregation and conformational effects in the active material, as implied by the relatively retained PL and PLQY.

**Fig. 3:**
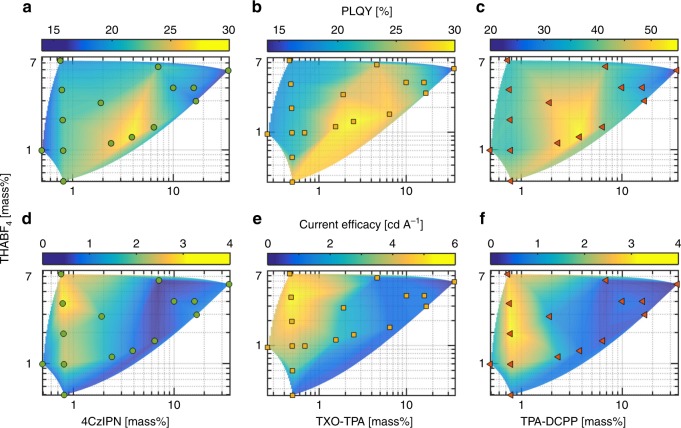
Optimization of active material. Surface plots of the photoluminescence quantum yield (PLQY) of the active-material films (**a**–**c**), and the current efficacy of devices (**d**–**f**), as a function of the concentration of the thermally activated delayed fluorescence (TADF) guest (*x*-axis) and the THABF_4_ electrolyte (*y*-axis). The TADF guest is: 4CzIPN (**a**, **d**), TXO-TPA (**b**, **e**), and TPA-DCPP (**c**, **f**). The markers indicate the measurement data, and the surface plots were fabricated as a guide to the eye by interpolation using the function griddata in Matlab. The thickness of the active-material films was 100 nm, the excitation wavelength for the PLQY measurement was 300 nm, and the drive current density for the device measurement was 100 A m^−2^.

The observed strong influence of both the guest concentration and the surrounding matrix on the PLQY and the PL spectrum in Fig. 2 inspired a systematic study on the optimum composition of the LEC active material, as summarized in the surface plots in Fig. 3. Figure 3a–c present the results for the PLQY, for which the optimum value for the guest concentration was in the range of 3–5 mass%, while the preferred value for the electrolyte concentration was 1.3 mass%. This parameter selection resulted in a PLQY of 28% for the active material based on 4CzIPN, 30% for TXO-TPA, and 54% for TPA-DCPP, i.e., slightly higher values than those of the more guest- and electrolyte-rich active materials presented in Fig. 2. It is, however, important to remember that the active-material composition for the maximum PLQY not necessarily translates into a peak performance when the active material is implemented in an LEC device, since specific device-operation effects such as charge trapping and electrochemical doping can markedly alter the preferred composition of the active material.

We have fabricated and characterized LEC devices with an indium-tin-oxide (ITO)/poly(3,4-ethylenedioxythiophene):poly(styrene-sulfonate) (PEDOT:PSS)/active-material/Al architecture, and Fig. 3d–f presents the measured peak current efficacy (at 100 A m^−2^ drive current density) as a function of the active-material composition. We find that the active-material composition for optimum LEC performance was notably different from that of peak PLQY, with the THABF_4_ electrolyte concentration being higher at 3.8 mass% while the preferred guest concentrations were markedly lower at 0.8 mass% for 4CzIPN and TPA-DCPP and 0.5 mass% for TXO-TPA. The lower guest concentration for optimized EL originates in that charge trapping contributes to the host-to-guest energy transfer in EL but not in PL. We also call attention to that the device-optimized low guest-concentration LECs featured a blue-shifted emission compared with the guest-richer LECs, and that, e.g., the optimized low guest-concentration 4CzIPN LEC featured cyan emission centered at ~505 nm, whereas the more guest-rich 4CzIPN LEC exhibited green emission centered at ~520 nm.

**Fig. 4:**
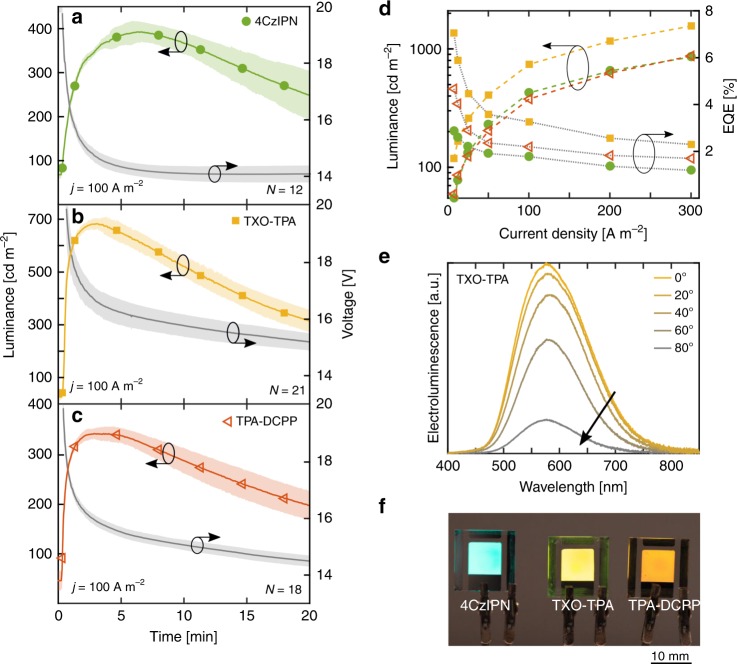
Device performance of optimized host-guest light-emitting electrochemical cells (LECs). **a**–**c** The temporal evolution of the luminance (left *y*-axis) and the voltage (right *y*-axis) during constant-current driving of the optimized ITO/PEDOT:PSS/active-material/Al devices, with the thermally activated delayed fluorescence (TADF) guest identified in the insets. The optimized guest concentration and active-material thickness were 0.5 mass% and 130 nm (**a**, **c**) and 0.8 mass% and 140 nm (**b**). The electrolyte concentration was 3.8 mass%, and the drive current density was 100 A m^-2^. **d** The peak luminance (left *y*-axis) and external quantum efficiency (EQE) (right *y*-axis) as a function of current density for the LEC devices, with the TADF guest being 4CzIPN (solid green circles), TXO-TPA (solid orange squares) and TPA-DCPP (open red triangles). **e** The steady-state electroluminescence (EL) spectrum at different viewing angles for the optimized TXO-TPA based LEC. The data were recorded after ~5 min driving at a current density of 100 A m^−2^, i.e., close to the peak luminance. The arrow indicates increased viewing angle. **f** Photograph of the uniform 8 × 8 mm^2^ emission area of three TADF-LECs during driving at 100 A m^−2^.

Figure 4a–c displays the temporal evolution of the luminance (left *y*-axis) and the drive voltage (right *y*-axis) for the optimized devices when driven by a constant-current density of 100 A m^−2^. These TADF-LECs had been further optimized with respect to the active-material thickness, and the best performance was obtained with a thickness of 130 nm (4CzIPN and TPA-DCPP) and 140 nm (TXO-TPA). The solid line and symbols depict the median performance with the number of investigated devices (*N*) being 12 for 4CzIPN, 21 for TXO-TPA, and 18 for TPA-DCPP; while the hero device performance at a number of different drive current densities is presented in Supplementary Fig. 2. The shaded area in Fig. 4a–c indicates the average deviation from the median, and the low variability in the data demonstrates high device repeatability and fabrication robustness.

We find that all three TADF-LECs exhibit the characteristic LEC features, with increasing luminance and decreasing voltage during the initial ~5 min of constant-current operation. The increasing luminance originates in a more balanced hole and electron injection when the p–n junction forms in the active material, while the lowering voltage results from an improved injection and transport during electric-double-layer formation and electrochemical doping, respectively. These observations are in line with that the blend host and the three TADF guests exhibit balanced electrochemical p-type and n-type doping capacity, as deduced from the voltammograms displayed in Fig. 1a–f. We tentatively assign the high steady-state voltage at high luminance to the existence of non-filled traps in the p–n junction region^11^, but also emphasize that significant luminance was detected at a much lower drive voltage of 4 V. We find that an increase of the electrolyte concentration from the optimum value of 3.8 mass% resulted in a slightly lowered drive voltage and a faster turn-on, but at the expense of lower efficiency and stability; while a decrease of the electrolyte concentration resulted in slower turn-on, higher driving voltage and decreased efficiency.

Importantly, the three optimized TADF-LECs delivered a very good light-emission performance. For the hero devices driven by 100 A m^−2^ (see Supplementary Fig. 2), the 4CzIPN LEC exhibited cyan luminance with 430 cd m^−2^ at 1.8% EQE and 4.3 cd A^−1^ current efficacy, the TXO-TPA device displayed yellow emission with 740 cd m^−2^ at 3.3%/7.4 cd A^−1^, and the TPA-DCPP LEC emitted orange light with 380 cd m^−2^ at 2.2%/3.8 cd A^−1^. Figure 4d presents the peak luminance (left-*y*-axis, dashed lines) and the EQE at peak luminance (right *y*-axis, solid lines) as a function of the current density, and demonstrates that a high luminance well above 1000 cd m^−2^ is attainable from the TADF-LECs but that the penalty is a lowered efficiency. Nevertheless, we call attention to that the TXO-TPA LEC (solid orange squares) exhibited a very high EQE of 7.0% and a current efficacy of 16.0 cd A^−1^ (Supplementary Fig. 3a) at a significant luminance of 120 cd m^−2^. The turn-on time to a luminance above 100 cd m^−2^ (at 100 A m^−2^ current density) was relatively fast at 20–25 s for all three TADF-LECs, and these turn-on kinetics could be further improved by simply increasing the drive current (or increasing the electrolyte concentration). As the turn-on of an LEC is directly dependent on ion redistribution to the electrode interfaces, this implies that the bulky ionic-liquid ions are highly mobile by being well-blended into the active material.

The steady-state electroluminescence (EL) spectrum as a function of viewing angle for the TADF-LECs is displayed in Fig. 4e (TXO-TPA), Supplementary Fig. 3b (4CzIPN), and Supplementary Fig. 3c (TPA-DCPP). We observe that the EL spectrum is relatively invariant to the viewing angle for all three optimized TADF-LECs, which implies that emission-shifting cavity effects are essentially non-existent for this specific device architecture and active-material thickness^58–60^. This conclusion is supported by the luminous intensity distribution depicted in Supplementary Fig. 4, which shows that the three TADF-LECs exhibit a luminous intensity that essentially scales with the cosine of the viewing angle; in other words, all three optimized TADF-LECs feature a close-to-Lambertian shaped emission profile.

A comparison of the EL spectrum with the PL spectrum of the blend host (Fig. 1i, solid blue squares) and the PL spectrum of the optimized active material demonstrates that the host-to-guest transfer is complete in EL, and that the EL accordingly stems solely from the guest for all three TADF-LECs. The 4CzIPN LEC exhibits its EL peak at 505 nm (CIE *xy* coordinates = 0.31, 0.47), the TXO-TPA LEC at 580 nm (0.46, 0.50), and the TPA-DCPP LEC at 618 nm (0.54, 0.44). Figure 4f presents a photograph of three representative TADF-LECs during light emission when driven by a current density of 100 A m^−2^, and we call attention to the uniform emission from the entire 8 × 8 mm^2^ device area.

### Photophysical characterization

**Fig. 5:**
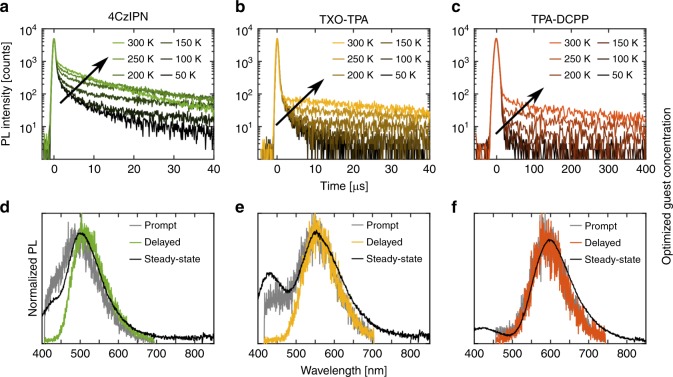
Transient photoluminescence (PL) decay and emission spectra. **a**–**c** The PL intensity transient as a function of temperature, and **d**–**f** the time-resolved PL spectra at 300 K, for the device-optimized active materials. The PL transients were measured with an excitation wavelength of 337 nm, while the steady-state PL spectra were excited at 300 nm. The film thickness was 100 nm, and the arrows in (**a**–**c**) indicate increasing temperature.

The guest molecules were selected for their capacity of TADF emission, but this capacity has up to now not been directly verified in the active material of our LEC devices. In fact, Dos Santos and co-workers^61^ and others^62^ reported that the efficiency of the TADF emission from a molecule is directly dependent on its conformational state, and that the physical environment of the molecule (e.g., in the LEC active material) accordingly will have a decisive role on its capacity for efficient TADF emission. With this in mind, we have recorded PL transients of the device-optimized active materials as a function of temperature, since the TADF-characteristic temperature-activated “delayed” transient, originating in the thermal activation of excitons from the “dark” T_1_ state to the emissive S_1_ state by RISC, only will be present in an emitter that is TADF active. Figure 5a–c reveals that the device-optimized active materials display a thermally activated delayed transient in addition to the prompt transient, and the conclusion must thus be that all three of the active materials indeed display the much desired TADF emission in LEC devices.

Figure 5d–f presents time-resolved PL spectra, which were recorded during the initial “prompt” period and during the “delayed” phase. A time-integrated “steady-state” PL spectrum (detected in a separate setup) is also included, and should comprise a combination of the prompt and delayed spectra. A comparison with the previously presented steady-state PL spectra for the host (Fig. 1i, solid blue squares) discloses that residual host emission centered at ~430 nm is apparent in both the prompt and steady-state PL spectra, but not in the delayed PL spectra. The lack of delayed host emission suggests that the triplets on the blend host are effectively transferred to the guest before emission, or alternatively that the host triplets have decayed as heat before the transfer. Importantly, the strong spectral resemblance of the prompt fluorescence and the delayed component (excluding the minor contribution from the host) implies that both originate from the same excited state, which yields further support for TADF emission via RISC from T_1_ to S_1_.

Supplementary Fig. 5 presents the corresponding PL transients as a function of temperature, and the time-resolved PL spectra at room temperature, for active materials with a higher guest (and electrolyte) concentration (see figure caption for details). The guest-rich active materials also display TADF emission, although the delayed component is of a lower magnitude than for the device-optimized materials. We further note that the host emission is completely absent in the guest-rich materials, implying complete host-to-guest energy transfer. We finally observe that the PL peaks are red-shifted by ~40–50 nm in comparison to the device-optimized active materials in Fig. 5, which suggests that guest aggregation is rather prominent in these guest-rich active materials.

We have estimated the singlet-triplet energy gap (∆*E*_ST_) with the difference in energy between the PL peak at 300 K (“the fluorescence peak”) and the delayed PL peak at 77 K (“the phosphorescence peak”, see Supplementary Fig. 6, and associated Supplementary Note 1). As expected ∆*E*_ST_ is very small for the optimized active materials at 25 meV for 4CzIPN, 105 meV for TXO-TPA, and 140 meV for TPA-DCPP. We have also calculated the different rate constants using the procedure outlined in ref. ^63^, and these detailed data for both the device-optimized and a guest-rich active material are presented in Supplementary Table 2. Interestingly, we find that the time constant for the delayed fluorescence component is much larger for the TPA-DCPP active material than for the other two materials, which is in agreement with that the rate constant for RISC transition is lowest and ∆*E*_ST_ largest for this material^28^. In this context, we remember that the PL data in Fig. 2 indicated a comparatively non-favorable environment in the active material for TPA-DCPP.

### Device analysis and outlook

Three well-established criteria for functional LEC operation are that the active material features significant and balanced p- and n-type doping capacity, that the active-material constituents are compatible and well-blended, and that all device constituents, including the electrolyte and the electrodes, feature a sufficient electrochemical stability in order to suppress undesired side reactions during the electrochemical operation. The CV study in Fig. 1 demonstrates that the PVK:OXD-7 blend host, as well as the three guest compounds, can be both p-type and n-type doped, which implies that balanced electrochemical doping will take place and result in the formation of the desired light-emitting p–n junction structure. This conclusion is supported by the observation of characteristic LEC transients in the form of an increasing luminance and a decreasing voltage during the initial LEC device operation in Fig. 4a–c. The high compatibility and good blending capacity of the active-material constituents are manifested in the exciplex emission from the blend host (Supplementary Fig. 1b) that depend upon the intimate mixing of the two host constituents, the efficient host-to-guest energy transfer in EL even at very low guest concentration below 1 mass% (Fig. 4e and Supplementary Fig. 3b, c), and the second-fast turn-on time of the LEC devices that imply that the ions are in close proximity to the host and guest. The electrochemical stability of the electrolyte was illustrated to be sufficient in Fig. 1h, while ref. ^64^ demonstrated that the ITO anode and the Al cathode are stabile in the voltage range in which the active materials are electrochemically doped. Thus, the developed device design fulfills the basic criteria for functional LEC operation.

In order to achieve efficient emission from a device void of expensive and rare metals, it is preferable if the active material exhibits TADF emission. That this feature was in effect in our LEC devices was demonstrated by the characteristic temperature-activated delayed transients in Fig. 5a–c and the essentially perfect overlap by the prompt and the delayed emission spectra in Fig. 5d–f. Further support for TADF emission from the LEC devices is provided by the measured values for the PLQY and the EQE and a related analysis. Specifically, the fraction of the electrically generated excitons that are passing an emissive state during the exciton lifetime can be estimated by:

1 $${{\Omega }}_{{\mathrm{ES}}} = {\mathrm{EQE}}/(\eta _{{\mathrm{rec}}} \times {\mathrm{PLQY}} \times \eta _{{\mathrm{out}}} \times X_{{\mathrm{loss}}})$$

where *η*_rec_ is the ratio of the number of exciton formation events within the device to the number of electrons flowing in the external circuit, *η*_out_ is the outcoupling efficiency of the device structure, and *X*_loss_ is a factor that represents the combined additional loss mechanisms due to, e.g., exciton–polaron quenching^65^.

For an LEC device with the p–n junction formed away from the electrode interfaces, it is reasonable with *η*_rec_ = 1, in consideration of that a minority carrier should recombine with the majority carrier during the passage of a doped region. The exact values for *η*_out_ and *X*_loss_ are not known, but in order to establish a lower limit for Ω_ES_, we selected high values of *η*_out_ = 0.3 and *X*_loss_ = 1, where the latter (somewhat naively)^66^ assumes that exciton–polaron losses are negligible. With this conservative estimate, Ω_ES_ is equal to 0.5 for the 4CzIPN LEC, 1.2 for TXO-TPA, and 0.4 for TPA-DCPP. A value above 1 for Ω_ES_ for the TXO-TPA LEC is not physically possible, but it is plausible that the measured value for the PLQY underestimates the “effective PLQY” during device operation, because the non-efficient host emission that is relatively prevalent during the measurement of the PLQY in the active material (Fig. 5d–f) is effectively eliminated during device operation because of charge trapping (see Fig. 4e and Supplementary Fig. 3b, c). Nevertheless, the finding of a Ω_ES_ value significantly above 0.25 for all three guest compounds yields additional support for that both the triplet and singlet excited states contribute to the light emission of the LEC devices, and that the LEC devices indeed emit with the TADF mechanism.

It is reasonable to assume that the charge transport during device operation is effectuated by the majority (>95 mass%) host, while the presented EL data demonstrate that the emission originates solely from the minority (<1 mass%) guest. The recorded data further suggest that the efficient host-to-guest transfer in the LEC devices is executed by a combination of FRET, Dexter transfer and charge trapping, but also that some differences exist between the different LECs. For the largest-gap 4CzIPN device, the low HOMO of the guest prohibits hole trapping so that it is only electrons that are effectively trapped during device operation. The smallest-gap TPA-DCPP featured the highest PLQY value in isolated form in dilute solution (Fig. 2c), which is notable in consideration of the energy-gap law that states that the non-radiative rate increases exponentially with decreasing energy gap^67,68^, but at the same time this emitter was found to be most sensitive to quenching aggregation/conformation effects in the host-guest solid-state material.

We further note that the peak in luminance significantly precedes the minimum in voltage for the TADF-LECs (Fig. 4a–c), which suggests that the exciton and doping-polaron populations are beginning to markedly overlap with increasing doping at longer operational times, and that exciton–polaron quenching is significant at steady-state operation^69,70^. This explanation implies that the luminance drop should be (partially) reversible following a rest time. Supplementary Figs. 7–9 and the related text in the Supplementary Note 2 present a systematic investigation towards this end, which indeed demonstrates that the initial luminance drop is completely reversible whereas a longer operation also results in a non-reversible loss of luminance. A recent study^11^ demonstrated that such detrimental exciton–polaron interactions can be significantly suppressed in host-guest LECs through the employment of balanced hole and electron traps (and balanced electron and hole mobility), and we remind that the herein studied TADF-LECs feature relatively imbalanced hole and electron traps; see Fig. 1h and Supplementary Table 1. We also note that the roll-off in the efficiency with increasing current is rather significant (see Fig. 4d) and speculate that this could be due to exciton–exciton quenching. More specifically, the combination of a low guest concentration (<1 mass%) and a long emission lifetime (Supplementary Table 2 shows that the delayed fluorescence component features an emission lifetime of 10–100 µs) suggests that exciton–exciton quenching can become severe at higher current densities.

It is further plausible that such exciton–polaron and exciton–exciton quenching interactions can result in the formation of highly energetic species that are prone to detrimental side reactions, and that these processes therefore also can limit the operational stability of the TADF-LECs. Accordingly, it seems plausible that a further improvement in the device efficiency and stability can be attained through the design and development of a TADF-active host:guest:electrolyte active material with more balanced electron and hole traps (at retained balanced electron and hole mobility), a higher guest concentration and a shortened emissive lifetime.

We have finally estimated the steady-state doping structure in the optimized active materials using the procedure outlined in Supplementary Note 3. It shows that all of the guest molecules and 15% of the host units are doped in the two doping regions sandwiching the undoped p–n junction at which excitons are formed, and we note with interest that the optimization resulted in the same steady-state doping structure for all three active-material systems.

## Discussion

We conclude by summarizing our most important findings. We demonstrate that it is possible to attain efficient and bright TADF emission from an LEC device, comprising a metal-free single-layer active material sandwiched between two air-stabile electrodes. This was accomplished by the introduction of an active material that comprises a majority polymeric blend host that allows for balanced electrochemical doping and electronic transport as well as for uniform film formation, an optimized concentration of a guest for complete host-to-guest energy transfer at minimized guest–guest aggregation and efficient TADF emission, and a relatively low concentration (3.8 mass%) of an electrochemically stabile ionic-liquid electrolyte for appropriate electrochemical doping. We specifically report on a yellow-emitting TADF-LEC that delivers a luminance of 120 cd m^−2^ at an EQE of 7.0%, corresponding to a current efficacy of 16 cd A^−1^, but also mention that the introduced generic approach allowed for realization of bright and efficient orange and cyan emission from two other similarly optimized TADF-LECs.

## Methods

### Materials and inks

A blend of the high-molecular weight polymer poly(9-vinylcarbazole) (PVK, *M*_w_ = 1.1 × 10^6^ g mol^−1^, 193.24 g mol^−1^ per repeat unit, Sigma-Aldrich) and the small molecule 1,3-bis[2-(4-tert-butylphenyl)-1,3,4-oxadiazo-5-yl]benzene (OXD-7, 478.58 g mol^−1^, Lumtec) in a 1:1 mass ratio was the host. Three different compounds were investigated for the guest: (i) 2,4,5,6-tetra(9H-carbazol-9-yl)isophthalonitrile (4CzIPN, 788.89 g mol^−1^, Lumtec)^22^, (ii) 2-[4-(diphenylamino) phenyl]-10,10-dioxide-9H-thioxanthen-9-one (TXO-TPA, 487.57 g mol^−1^, Lumtec)^28^, and (iii) 7,10-Bis(4-(diphenylamino)phenyl)-2,3-dicyanopyrazinophenanthrene (TPA-DCPP, 766.89 g mol^−1^, Lumtec)^51^. The electrolyte was tetrahexylammonium tetrafluoroborate (THABF_4_, 217.07 g mol^−1^, Sigma-Aldrich). All materials were used as received.

The master inks were prepared by separately dissolving the above materials in chlorobenzene in a concentration of 20 g L^−1^ by stirring on a magnetic hot plate at 323 K for > 4 h. The active-material inks were prepared by blending the master inks in a desired mass ratio, followed by stirring on a magnetic hot plate at 323 K for > 4 h. In total, more than 45 different active-material compositions were investigated.

### Material characterization

The ink-under-study was spin-coated (1000 rpm, 1000 rpm s^−1^, 60 s) on a carefully cleaned substrate, and thereafter dried at 343 K for >4 h. The thickness of the dry active-material film was measured with a profilometer (DekTak XT, Bruker). The optical transmission of the active-material film on a quartz substrate (thickness = 1 mm, Ted Pella) was measured with a spectrometer (C9920-02G, Hamamatsu Photonics). The absorbance was estimated as [1−transmission], thus neglecting the effects of reflectance. The steady-state photoluminescence (PL) spectrum and the PL quantum yield (PLQY) at 300 K of the films and solutions were measured with an integrated sphere connected to a spectrometer (C9920-02G, Hamamatsu Photonics). The PL spectrum at 77 K was recorded with a spectrofluorometer (JASCO FP-8600), by submerging the drop-cast film in liquid nitrogen. It is noted that the 77 K PL spectrum was recorded after a delay of 10 ms following excitation in order to selectively capture the phosphorescence. The 300 K PL spectra were measured on a 100-nm thick spin-coated films that were excited at 300 nm, and the 77 K PL spectra were measured on drop-cast films excited at 400 nm.

The transient PL emission was recorded on an active-material film on a Si substrate (Siegert Consulting) under vacuum by a streak camera (C4334, Hamamatsu Photonics), and using a nitrogen laser (Ken-X, Usho Optical System), delivering 800 ps excitation pulses with a wavelength of 337 nm at a repetition rate of 20 Hz, as the excitation source. For 4CzIPN and TXO-TPN, the prompt and delayed PL emission spectra were recorded in the streak time range of −1.5–1.5 μs and 1.5–40 μs, respectively. For TCA-DCPP, the prompt and delayed PL emission spectra were recorded in the streak time range of −10–10 μs and 10–400 μs, respectively. Note that the prompt PL was recorded with a higher temporal resolution.

Cyclic voltammetry (CV) was carried out on a three-electrode setup connected to a computer-controlled potentiostat (Autolab PGSTAT302, software: GPES). The working electrode comprised the material-under-study drop-cast on a Au-covered glass substrate, a Pt rod was the counter electrode, a Ag wire was the quasi-reference electrode, and 0.1 mol L^−1^ tetrabutylammonium hexafluorophosphate (TBAPF_6_, Sigma-Aldrich. The scan rate was 0.05 V s^−1^. Directly after each CV scan, a calibration scan was run with a small amount of ferrocene added to the electrolyte, so that the CV potentials could be reported vs. the ferrocene/ferrocenium ion (Fc/Fc^+^) reference potential. The reduction/oxidation onset potentials were defined as the intersection of the baseline with the tangent of the current at the half-peak-height. The CV preparation and characterization were executed within a N_2_-filled glove box ([O_2_] < 1 ppm, [H_2_O] < 0.5 ppm). Note that we employed the TBAPF_6_ ionic liquid as the electrolyte in the CV over the THABF_4_ ionic liquid that was used in the LEC devices since the former produced more repeatable CV traces for the TADF materials.

### Device fabrication and characterization

Indium-tin-oxide (ITO) coated glass substrates (ITO thickness = 145 nm, 20 Ω sq^−1^, Thin Film Devices) were cleaned by sequential 20 min ultrasonic treatment in detergent (Extran MA 01, Merck), distilled water, acetone (VWR), and isopropanol (VWR), drying at 393 K for >4 h, and UV-ozone exposure for 20 min. Poly(3,4-ethylenedioxythiophene):poly(styrene-sulfonate) (PEDOT:PSS, Clevios PVP AI 4083, Heraeus) was spin-coated (4000 rpm, 1000 rpm s^−1^, 60 s) on top of the ITO, and dried at 393 K for >2 h. The thickness of the dry PEDOT:PSS film was 35 nm. The active-material ink was spin-coated on top of the PEDOT:PSS, and dried at 343 K for >3 h. The thickness of the active material could be controlled by the spin-coating parameters, and the optimized thickness was 130 nm for 4CzIPN and TPA-DCPP (1000 rpm, 1000 rpm s^−1^, 60 s), and 140 nm for TXO-TPA (800 rpm, 1000 rpm s^−1^, 60 s). A 100 nm thick Al cathode was deposited on top of the active material by thermal vacuum evaporation at a base pressure below 2 × 10^−6^ mbar. The emission area was defined by the overlap between the Al top cathode and the ITO bottom anode, and the employment of two different shadow masks during the Al evaporation resulted in a small emission area of 2 × 2 mm^2^ and a large emission area of 8 × 8 mm^2^. All of the above procedures, with the exception of the PEDOT:PSS deposition, were carried out in two interconnected N_2_-filled glove boxes ([O_2_] < 1 ppm, [H_2_O] < 0.5 ppm).

The LEC devices were driven and measured with a computer-controlled current–voltage–luminance system (OLED Lifetime Tester M6000, McScience), with the luminance measured in the forward direction. The devices were characterized within 48 h of fabrication. The angle-dependent electroluminescence (EL) spectrum and intensity were measured with a custom-built spectroscopic goniophotometer, essentially comprising a fiber-optic CCD-array spectrometer (Flame-S, Ocean Optics) and a stepper motor, controlled with a LabVIEW virtual instrument program^58^. The photographs of the LECs during light emission were captured with a digital single-lens reflex camera (Canon EOS 60D) equipped with a telephoto lens (150 mm F2.8 EX DG HSM, Sigma), with the recorded colors slightly adjusted to better reflect the perception of the human eye.

## Supplementary information

Supplementary Information

## Data Availability

Data available on reasonable request from the authors.
