# Supplementary material for: Thermally activated delayed fluorescence with 7% external quantum efficiency from a light-emitting electrochemical cell
Source: Nat Commun. 2019 Nov 22;10:5307. doi: 10.1038/s41467-019-13289-w (PMC6874610; doi:10.1038/s41467-019-13289-w)
Supplement: Supplementary file 1 — Supplementary Information [file 41467_2019_13289_MOESM1_ESM.pdf]

Supplementary Information

Thermally Activated Delayed Fluorescence with 7 % External Quantum Efficiency from a Light-Emitting Electrochemical Cell

Petter Lundberg[1], *Youichi Tsuchiya*[2,3], *E. Mattias Lindh*[1], *Shi Tang*[1,6], *Chihaya Adachi*[2,3,4,5], *and Ludvig Edman*[1,6,*]

[1] The Organic Photonics and Electronics Group, Umeå University, SE-901 87 Umeå, Sweden

[2] Center for Organic Photonics and Electronics Research (OPERA), Kyushu University, 744 Motooka, Nishi-ku, Fukuoka 819-0395, Japan.

[3] JST, ERATO, Adachi Molecular Exciton Engineering Project, Kyushu University, 744 Motooka, Nishi-ku, Fukuoka 819-0395, Japan.

[4] Department of Chemistry and Biochemistry, Kyushu University, 744 Motooka, Nishi-ku, Fukuoka 819-0395, Japan.

[5] International Institute for Carbon Neutral Energy Research (WPI-I2CNER), Kyushu University, 744 Motooka, Nishi-ku, Fukuoka 819-0395, Japan.

[6] LunaLEC AB, Linnaeus väg 24, SE-901 87 Umeå, Sweden.

* Corresponding author e-mail: ludvig.edman@umu.se

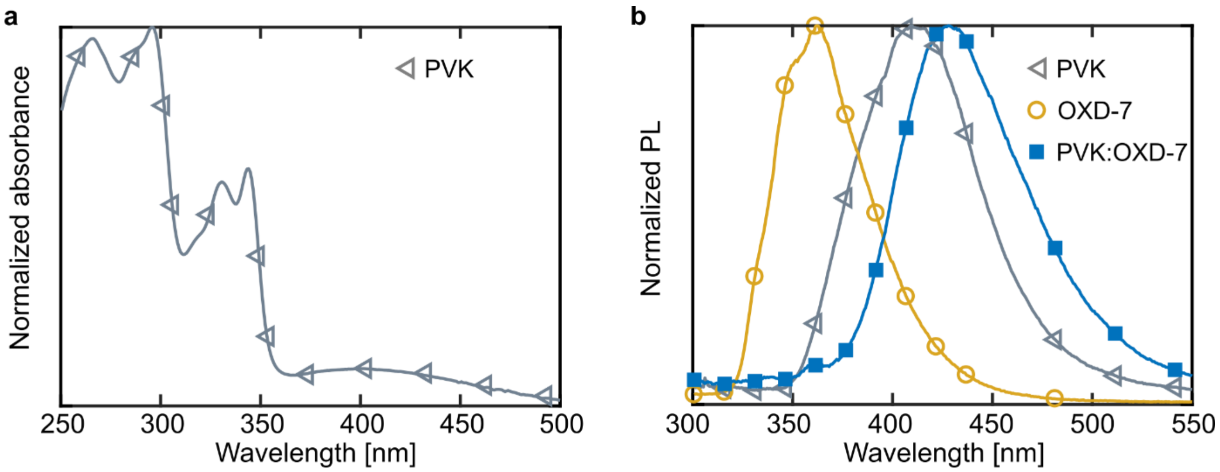

***Supplementary Figure 1**. Optical characterization of the host constituents and the blend host. **a** The normalized absorbance spectrum of a PVK film. **b** The normalized photoluminescence (PL) spectra of thin films of PVK (open gray triangles), OXD-7 (open yellow circles), and the PVK:OXD-7 blend-host (solid blue square). The film thickness was 100 nm, and OXD-7 was dispersed in poly(styrene) at 10 mass%. The PL excitation wavelength was 280 nm.*

Supplementary Table 1. *The highest occupied molecular orbital (HOMO) and lowest unoccupied molecular orbital (LUMO) energy levels of the host and guest compounds, and the electron and hole trap levels ($E_{\mathrm{trap}}^{\mathrm{n}}$ and $E_{\mathrm{trap}}^{\mathrm{p}}$) for the three host-guest systems.*

**Compound**	**HOMO [eV]**	**LUMO [eV]**	$E_{\mathrm{trap}}^{\mathrm{p}}$ **[eV]**	$E_{\mathrm{trap}}^{\mathrm{n}}$ **[eV]**
**PVK**	-5.6	-2.5	-	-
**OXD-7**	-6.3	-2.7	-	-
**PVK:OXD-7**	-5.6	-2.7	-	-
**4CzIPN**	-5.6	-3.4	0.0	-0.7
**TXO-TPA**	-5.3	-3.4	-0.3	-0.7
**TPA-DCPP**	-5.2	-3.4	-0.4	-0.7

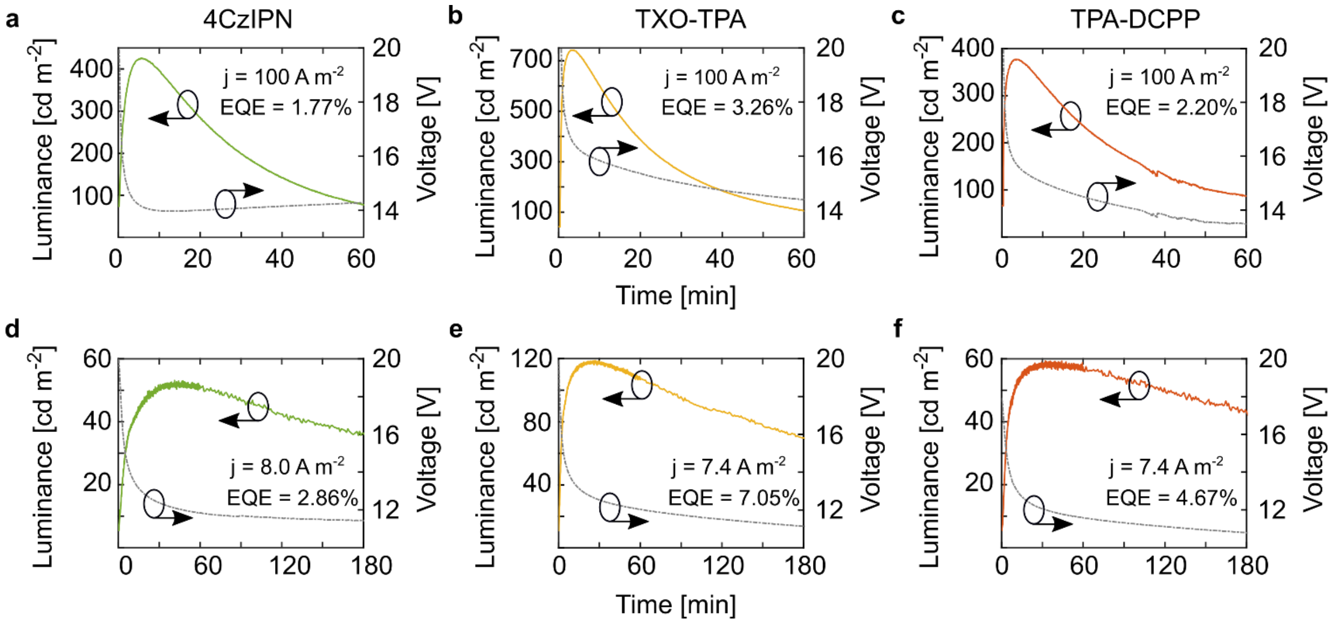

Supplementary Figure 2. *Device performance of the optimized host-guest light-emitting electrochemical cells (LECs). The temporal evolution of the luminance (left y-axis) and the voltage (right y-axis) during constant-current driving of optimized ITO/PEDOT:PSS/active-material/Al LECs, with the thermally activated delayed fluorescence (TADF) guest being: 4CzIPN (**a**, **d**), TXO-TPA (**b**, **e**) and TPA-DCPP (**c**, **f**). The guest concentration and the active-material thickness were: 0.5 mass% and 130 nm (**a**, **d**, **c**, **f**) and 0.8 mass% and 140 nm (**b**, **e**). The electrolyte concentration was 3.8 mass%. The first row (**a**-**c**) presents hero devices driven by a current density of 100 A m^{-2}, while the second row (**d**-**f**) shows the highest recorded efficiency for each TADF-LEC, with the drive current density specified in the inset.*

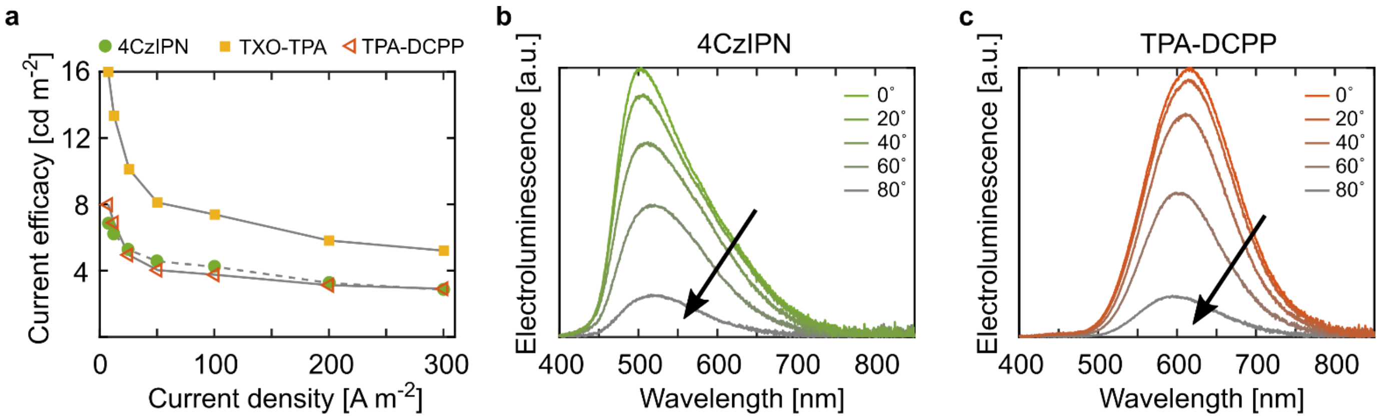

Supplementary Figure 3. *Device performance of the optimized light-emitting electrochemical cells (LECs).* ***a*** *The current efficacy as a function of current density for the ITO/PEDOT:PSS/active-material/Al LEC, with the thermally activated delayed fluorescence (TADF) guest being 4CzIPN (solid green circles), TXO-TPA (solid yellow squares) and TPA-DCPP (open red triangles).* ***b-c*** *The electroluminescence spectrum at different viewing angles for the ITO/PEDOT:PSS/active-material/Al LEC, with the TADF guest being 4CzIPN (**b**) and TPA-DCPP (**c**). The data were recorded at peak luminance, and the arrow indicates increased viewing angle.*

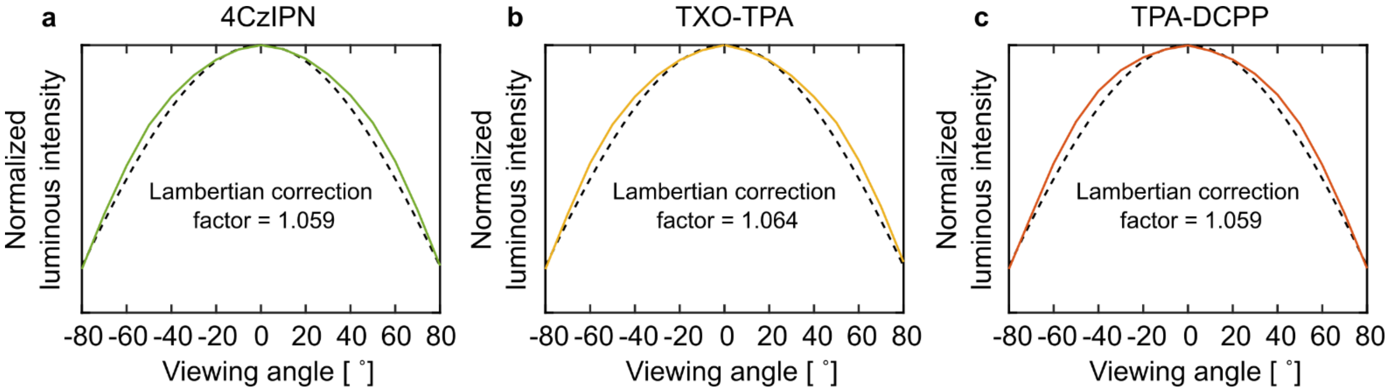

Supplementary Figure 4. *The luminous intensity as a function of viewing angle. The luminous intensity distribution for the optimized ITO/PEDOT:PSS/active-material/Al light-emitting electrochemical cells (LECs), with the thermally activated delayed fluorescence (TADF) guest being 4CzIPN (**a**), TXO-TPA (**b**) and TPA-DCPP (**c**). The data were recorded at peak luminance, and the black dashed line presents the luminous intensity distribution of an ideal Lambertian emitter.*

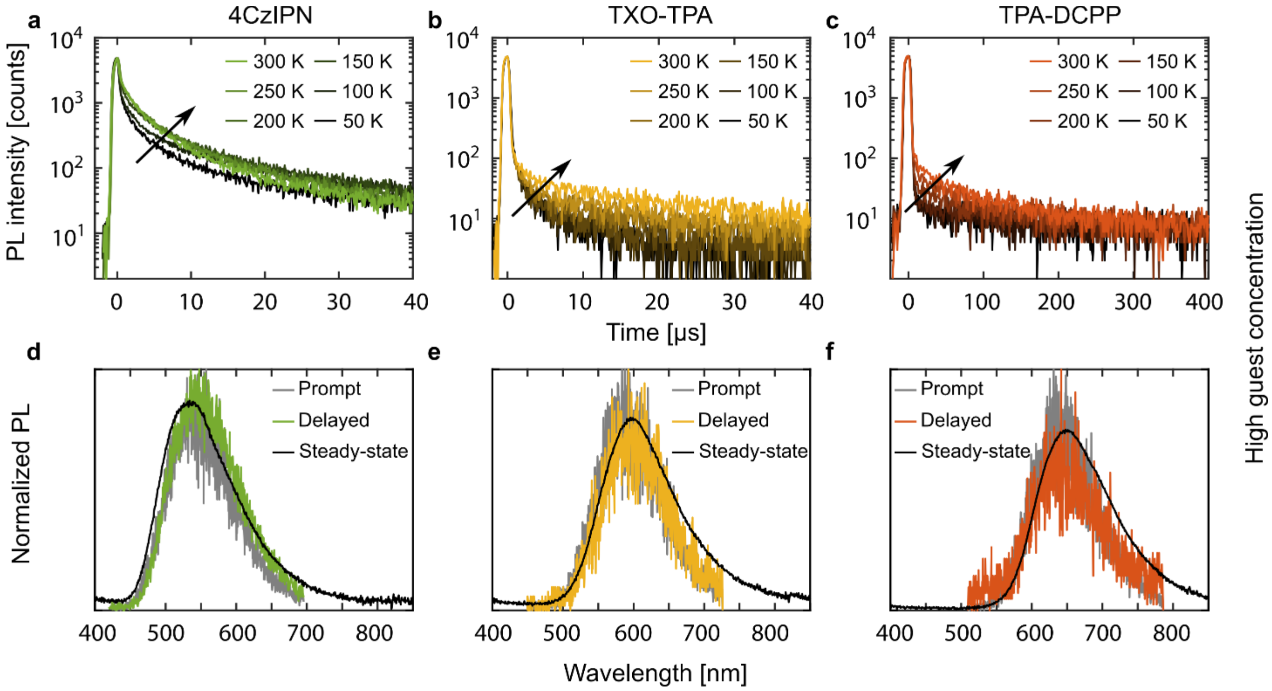

Supplementary Figure 5. *Transient photoluminescence (PL) decay and emission spectra. (**a**-**c**) The PL intensity transient as a function of temperature, and (**d**-**f**) the time-resolved PL spectra at 300 K, for guest-rich active materials comprising 7 mass% 4CzIPN (**a**, **d**), 4.6 mass% TXO-TPA (**b**, **e**) and 7 mass% TPA-DCPP (**c**, **f**). All active materials comprised 6.5 mass% THABF$_4$. The PL transients were measured with an excitation wavelength of 337 nm, while the steady-state PL spectra were excited at 300 nm. The film thickness was 100 nm, and the arrows in **a**-**c** indicate increasing temperature.*

Supplementary Table 2. *Photophysical properties of the active material. The singlet-triplet energy gap, the emission lifetimes, and the rate constants for the device-optimized active material, with the corresponding data for a higher guest-concentration active material in parenthesis.*

**Guest**	**ΔE_{ST} [meV]**	**LEC active material**					
		**Lifetime**		**Rate constant**			
		**τ_{PF} [ns]**	**τ_{DF} [µs]**	**k_r^S [s^{-1}]**	**k_{ISC} [s^{-1}]**	**k_{RISC} [s^{-1}]**	**k_{nr}^T [s^{-1}]**
**4CzIPN**	25 (0)	30 (17)	11 (12)	4.0 (13) ×10^6	2.9 (4.5) ×10^7	3.4 (2.2) ×10^5	4.7 (3.2) ×10^4
**TXO-TPA**	105 (90)	33 (20)	42 (25)	4.2 (13) ×10^6	2.6 (3.7) ×10^7	5.8 (5.2) ×10^4	1.6 (2.7) ×10^4
**TPA-DCPP**	140 (80)	21 (20)	465 (150)	1.3 (2.0) ×10^7	3.4 (3.0) ×10^7	2.6 (8.1) ×10^3	1.4 (3.4) ×10^3

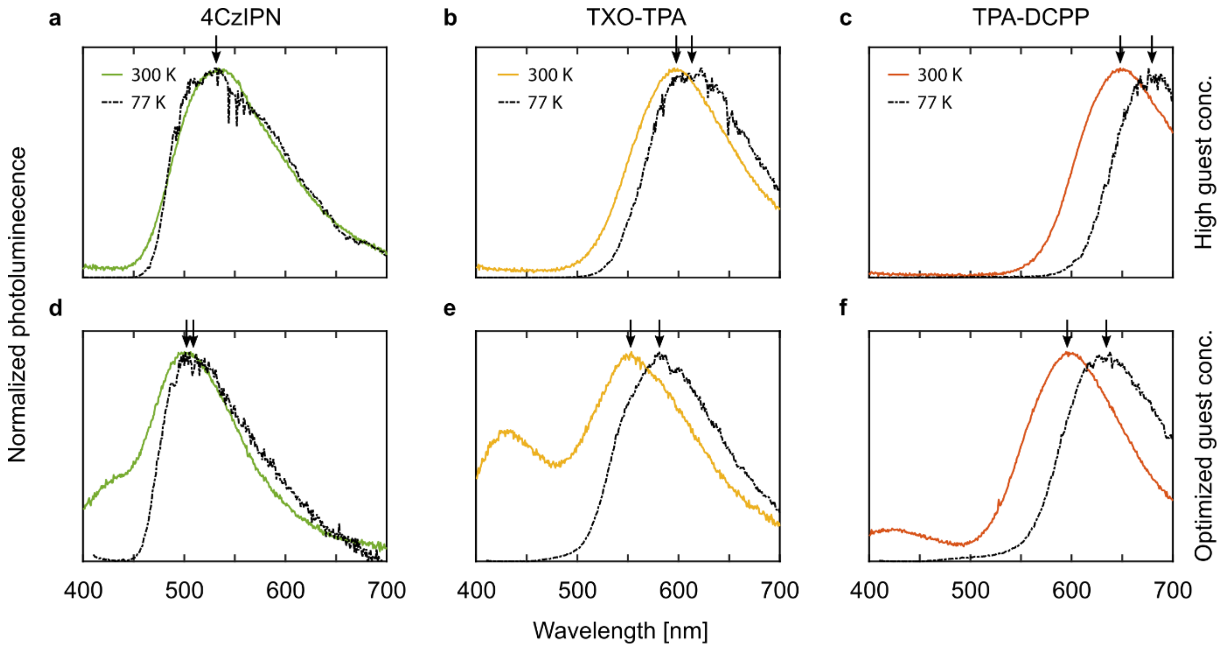

Supplementary Figure 6. *Fluorescence and phosphorescence spectra. The normalized photoluminescence (PL) spectrum at 300 K (solid line) and the normalized and delayed PL spectrum at 77 K (dash-dotted black lines) for the guest-rich active materials (**a**-**c**) and for the device-optimized active materials (**d**-**f**). The guest-rich active materials comprise 6.5 mass% THABF$_4$ and 7 mass% 4CzIPN (**a**), 4.6 mass% TXO-TPA (**b**) or 7 mass% (**c**) TPA-DCPP. The arrows indicate the PL peaks.*

Supplementary Note 1

The singlet-triplet energy gap (ΔE_{ST}) was estimated as the difference in energy of the PL peak at 300 K ("the fluorescence peak") and the delayed PL peak at 77 K ("the phosphorescence peak"), as presented in Supplementary Figure 6. The upper panel (Supplementary Figure 6a-c) presents data for the guest-rich active materials, and the lower panel (Supplementary Figure 6d-f) the corresponding data for the device-optimized active material, with the TADF guest identified above the graphs. The radiative lifetime of prompt fluorescence (τ_{PF}) and the radiative lifetime of delayed fluorescence (τ_{DF}), as well as the singlet radiative rate (k_r^S), the intersystem crossing rate (k_{ISC}), the reverse intersystem crossing rate (k_{RISC}), and the triplet non-radiative rate (k_{nr}^T), were calculated with the procedure detailed in Supplementary Reference 1 using the PL intensity transients at 300 K (see Figure 5 in main manuscript and Supplementary Figure 5; note that the calculation of τ_{PF} was done with PL transient data with a higher temporal resolution).

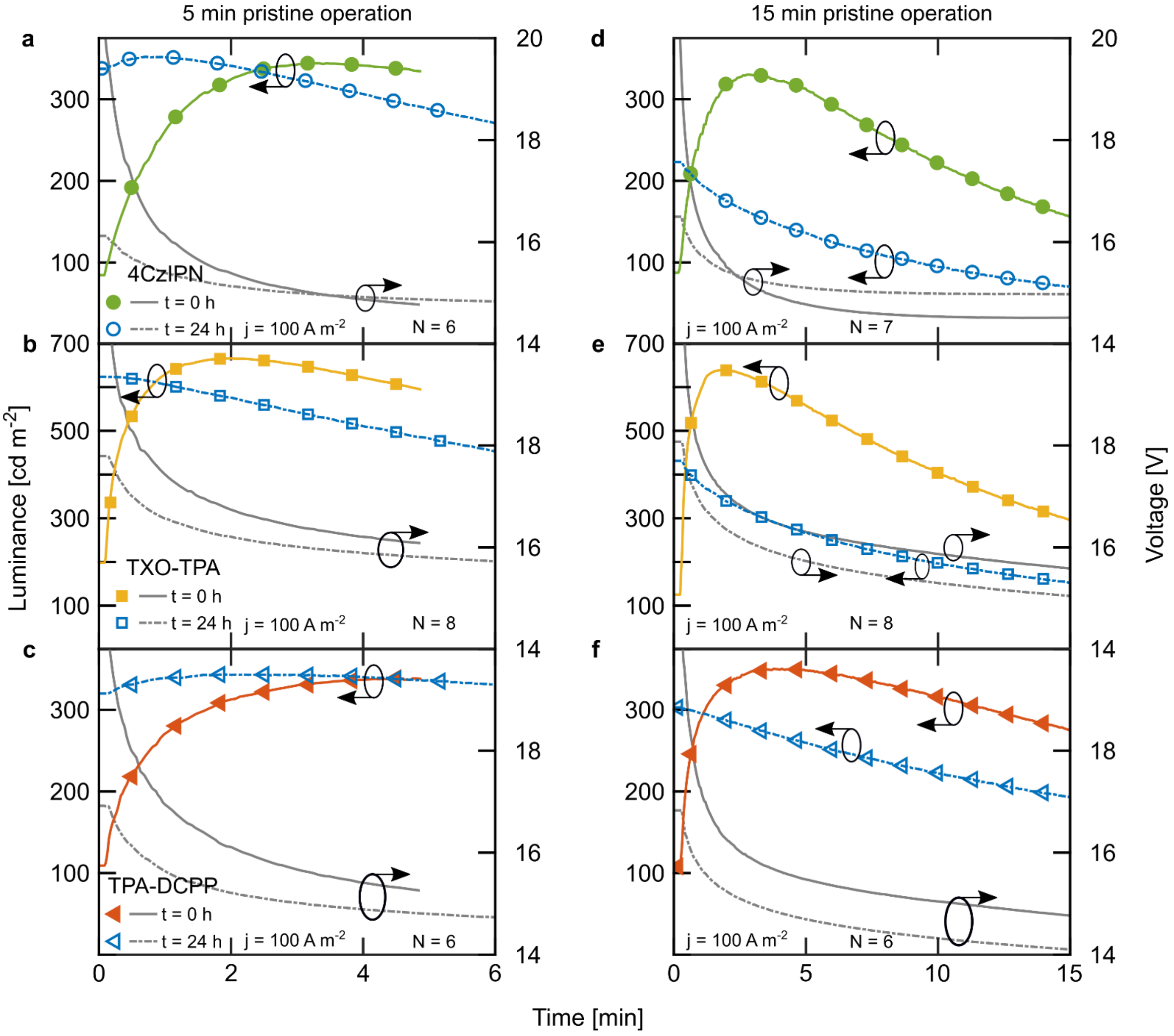

Supplementary Figure 7. *Pristine and recovered device performance. A comparison of the pristine performance (solid symbols and solid line) and the subsequent performance for the same device following 24 h of resting at open circuit (open symbols and dash-dotted line) for the optimized LEC devices, with the thermally activated delayed fluorescence (TADF) guest identified in the lower left inset.* ***a-c*** *present the data for pristine devices driven for 5 min, i.e. until close to peak luminance, while* ***d-f*** *show data for pristine devices driven for a longer time of 15 min. All devices were driven by a constant current density of 100 A* m^{-2}*. The presented data are the average for N investigated devices, with the value for N identified in the lower inset in each graph.*

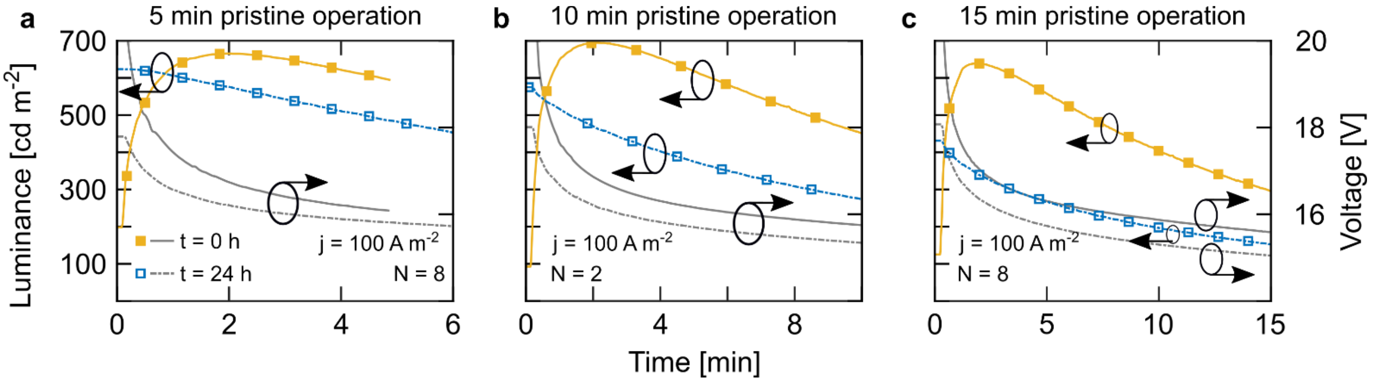

Supplementary Figure 8. *Pristine and recovered device performance. A comparison of the pristine (solid symbols and solid line) and the subsequently rested performance following 24 h at open circuit (open symbols and dash-dotted line) for the optimized TXO-TPA LEC as a function of the initial pristine operation time, as identified above the graphs. All devices were driven by a constant current density of 100 A m*$^{-2}$*. The presented data are the average for N investigated devices, with the value for N identified in the lower inset in each graph.*

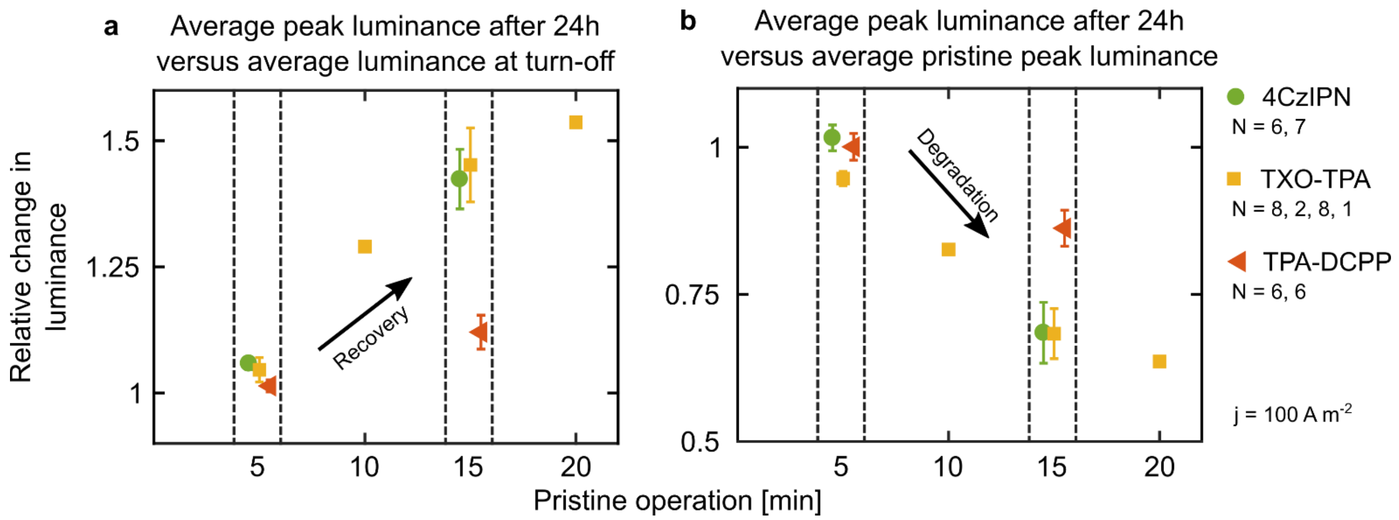

Supplementary Figure 9. *Performance recovery as a function of pristine operation time. The ratio between the peak luminance for a device rested for 24 h and the turn-off (**a**) or peak (**b**) luminance for the same device during its pristine operation as a function of pristine operation time. All devices were driven by a constant current density of 100 A m*$^{-2}$*. The presented data are the average for N investigated devices, with the values for N identified to the right of the graphs, and with the error bars representing the standard deviation.*

Supplementary Note 2

The reversibility of the device performance was investigated by first driving pristine devices at constant current for a set time (between 5 and 20 min), then resting the device for 24 h at open circuit, and finally recording the luminance and voltage transients a second time at the same constant current. Supplementary Figures 7-8 show that the drive voltage is consistently starting at a slightly lower value during the second run (compare dash-dotted line with solid line), which implies that either the first pristine operation has resulted in faster ion-redistribution kinetics or that some minor doping remains in the active material following one day of resting at open circuit. The situation for the luminance is more complex, since the performance of the rested devices depends strongly on the operational time of the pristine device, with a short pristine operational time of 5 min resulting in an essentially retained peak luminance while a longer operational lifetime of 15 min is concomitant with a drop in the peak luminance.

To distinguish between reversible and non-reversible changes induced by the initial pristine operation, we present in Supplementary Figure 9 the peak luminance during the second run normalized by the luminance of the pristine device during the first run at turn-off (Supplementary Figure 9a) and at peak luminance (Supplementary Figure 9b); these data are presented as a function of the pristine operational time. Supplementary Figure 9a thus provides a measure of the *reversible* recovery of the final luminance at the end of the first operational cycle, whereas Supplementary Figure 9b contributes with information on *non-reversible* effects on the peak luminance. We find that a 5 min initial operation of the pristine devices results in a complete reversibility of the peak luminance. With increasing pristine operational time, this situation changes and we observe a significant reversible recovery of the final luminance in the pristine run (Supplementary Figure 9a) but also a permanent degradation in the form of a lowered peak luminance (Supplementary Figure 9b).

Our conclusion is thus that the observed drop in luminance during the operation of pristine TADF-LECs originate in a combination of reversible and non-reversible effects. Based on preceding studies in the field, we suggest that the reversible recovery is due to a spatial separation of the doping/polaron and exciton populations during the off period, which results in lowered exciton-polaron quenching and increased efficiency. We further speculate that the high-energy species that is the result of the exciton-polaron and exciton-exciton interactions is (at least partially) the cause of the non-reversible change of the peak luminance via a degradation of the luminance capacity of the TADF emitter.

Supplementary Note 3 - Estimating the steady-state doping structure

Supplementary Reference 2 derived Supplementary Equation (1) for the calculation of the number of ions (N_{ions}) in a host-guest LEC required for the doping of all of the guest molecules and y % of the host molecules in the doped transport regions sandwiching the p-n junction, with the latter being undoped and occupying x % of the active-material thickness:

$$N_{\mathrm{ions}} = (1 - x) \cdot \left(N_{\mathrm{guest}} + y \times N_{\mathrm{host}}\right) \quad (1)$$

N_{guest} and N_{host} represent the total number of guest and host molecules, respectively, in the active material. The doping of all of the guests in the n-type/p-type transport region is equivalent to the filling of all of the electron/hole traps.

For 1 g of the optimized active material, we have: m_{THABF4} = 0.038 g, m_{guest} = 0.008/0.005 g, m_{host} = 0.954/0.957 g. The tabulated values for the molar mass are: M_{THABF4} = 217.07 g mol^{-1}, M_{4CzIPN} = 788.89 g mol^{-1}, $M_{\mathrm{TXO\text{-}TPA}}$ = 487.57 g mol^{-1}, $M_{\mathrm{TPA\text{-}DCPP}}$ = 766.89 g mol^{-1}, M_{host} (average of the PVK:OXD-7 constituents) = 336 g mol^{-1}. Combining these values, we get the following numbers for the different constituents in 1 g of optimized active material: N_{ions}= 3.5 × 10^{-4} mol, N_{guest}= 1.0 × 10^{-5} mol (deviation ≤ 2.5× 10^{-7} mol), and N_{host} = 2.8 × 10^{-3} mol.

By setting x = 0.2 and inserting the above number values into Supplementary Equation (1), we obtain y = 0.15 for all three systems. In other words, we find that the optimized LECs feature a steady-state doping structure in which all guest traps and 15 % of the host units are doped in the doped transport regions surrounding the light-emitting (and undoped) p-n junction.

Supplementary References

1. Masui K, Nakanotani H, Adachi C. Analysis of exciton annihilation in high-efficiency sky-blue organic light-emitting diodes with thermally activated delayed fluorescence. *Org Electron* **14**, 2721-2726 (2013).

2. Tang S*, et al.* Design rules for light-emitting electrochemical cells delivering bright luminance at 27.5 percent external quantum efficiency. *Nat. Commun.* **8**, 1190 (2017).
